# Editorial: Exploring burrowing in biological and robotic systems

**DOI:** 10.3389/frobt.2025.1730533

**Published:** 2025-12-05

**Authors:** Yasemin Ozkan-Aydin

**Affiliations:** University of Notre Dame, Notre Dame, IN, United States

**Keywords:** agricultural monitoring, space exploration in regolith, search and rescue operations, ecological analysis, shape-morphing, anisotropic friction, soft-bodied animals

Myriad strategies have been developed for a wide range of biological systems to successfully navigate and penetrate various challenging substrates, including soft, cohesive soil, heterogeneous granular media, and hard or stiff surfaces. The superior performance of these natural burrowers offers a compelling source of inspiration for researchers developing robotic systems for subterranean tasks. Potential applications span a diverse range of fields, including agricultural monitoring, space exploration in regolith, search and rescue operations, and ecological analysis. However, the creation of human-made subterranean systems faces a challenging set of problems, primarily due to the high resistive drag and lift forces encountered underground. To overcome these challenges, a deeper understanding of the biomechanics of successful biological burrowing is necessary. This Research Topic is dedicated to furthering our knowledge of biological burrowing strategies and morphologies and translating them into bio-inspired technologies that enable effective locomotion in complex environments. The five papers included here collectively build a cohesive narrative, charting the course from foundational theory to novel physical implementations.

The greatest constraint in subterranean robotics is the high energy cost associated with displacing or compacting soil. Rigid robotic systems (those designed primarily to drill or excavate) often fail to achieve the energy efficiency and adaptability of their natural counterparts. This is where soft robotics and compliant biological systems offer the best strategic advantage. By utilizing principles of shape-morphing, radial expansion, and anisotropic friction, many biological burrowers manage to manipulate local substrate forces, effectively turning a highly resistive environment into one they can navigate with ease. The goal is to precisely capture these principles, moving beyond brute force toward intelligent, low-energy subterranean locomotion.

This ambitious goal demands a thorough deconstruction of the biomechanics involved before we can successfully build and test prototypes. The first two papers in this Research Topic provide the necessary scaffolding for future work by establishing this foundational theoretical framework. In their comprehensive review, “*Fundamentals of burrowing in soft animals and robots*” (Dorgan and Daltorio), the authors systematically dismantle the complex act of burrowing into four universally applicable component challenges. They identify the necessity to create space within the solid substrate (whether through excavation, fracture, compression, or fluidization); the requirement for low-resistance locomotion using non-rigid kinematics like peristalsis or unbending; the essential need for generating thrust through effective anchoring (via anisotropic friction or radial expansion); and, finally, the integration of sensing and navigation to adapt the burrow path in real time. Their emphasis on soft-bodied burrowers is key, as these animals exemplify low-energy, minimal-disturbance interaction with the substrate.

Moving the field from biomechanical understanding toward practical engineering requires defining the obstacles ahead. Addressing the engineering challenges inherent in the biomechanical findings, “*Grand challenges for burrowing soft robots*” (Le et al.) shifts the focus squarely to the implementation side, exploring the specific role of soft materials in creating these highly adaptable robotic systems. The authors champion the biological principle of minimal environmental disturbance during penetration, a key differentiator from conventional mechanical diggers. They provide a high-level roadmap by conceptually dividing the burrowing process into two major stages: submerging (entry from the surface) and subterranean locomotion (travel underground). Importantly, they push the frontier by examining biological mechanisms for friction reduction, such as the use of material discharge like mucilage secretion. This critical challenge informs a new design philosophy: the robot must actively manipulate the surrounding media to reduce resistance.

With the challenges clearly defined and the soft-robotics mandate established, the remaining three original research papers offer innovative physical solutions, primarily focusing on the notoriously difficult area of granular media.

For instance, “*Mole crab-inspired vertical self-burrowing*” (Treers et al.) provides a tangible example, tackling the critical anchoring challenge identified in the reviews using a novel legged system inspired by the *Emerita analoga*. The resulting EMBUR robot achieves superior performance through the embedded intelligence of its leg design, which combines a sweeping leg trajectory with a specialized compliant fabric to create a powerful anisotropic force response. This is the physical realization of efficient anchoring, maximizing downward thrust while minimizing drag on the recovery stroke. Furthermore, the work successfully utilizes granular Resistive Force Theory (RFT) as a reduced-order model, demonstrating how theoretical physics can directly validate bio-inspired mechanical design.

The concept of maximizing anisotropy is then elegantly distilled in “*Efficient reciprocating burrowing with anisotropic origami feet*” (Kim et al.). This paper presents a beautiful, minimalist solution to the locomotion and anchoring problems. Instead of relying on complex, multi-actuator systems, the design uses foldable origami feet that passively induce the necessary anisotropic friction. With a single actuator applying only symmetric linear motion, the robot achieves highly efficient, directed burrowing, validating the power of leveraging smart material mechanics—a key theme from the review papers—to achieve complexity of motion with simplicity of actuation.

The narrative culminates by applying these concepts to one of the most extreme environments: submerged granular media. “*Burrowing and unburrowing in submerged granular media through fluidization and shape-change*” (Nayak et al.) presents a system that addresses the double challenge of both sinking and rising. Drawing inspiration from the razor clam’s brilliant strategy, the robotic system employs water-jet-based fluidization for its descent, drastically reducing drag. For the crucial, often-neglected problem of unburrowing (rising), the robot utilizes an untethered, soft, inflatable bladder that undergoes periodic radial expansion, a direct parallel to the soft-robot principles of anchoring and shape-morphing. This work is groundbreaking for applications in marine research, archaeology, and seabed infrastructure.

This Research Topic clearly demonstrates that the future of subterranean robotics lies in the symbiotic intersection of biology, material science, and engineering mechanics. These five papers take us from defining the four fundamental challenges and identifying the grand challenges of soft materials to creating specific, highly efficient hardware solutions that leverage anisotropic forces in granular media and fluidization in underwater environments. This Research Topic provides the essential tools, models, and design philosophies to drive the next-generation of robust, efficient, and truly autonomous subterranean systems.

